# Redox Imbalance and Morphological Changes in Skin Fibroblasts in Typical Rett Syndrome

**DOI:** 10.1155/2014/195935

**Published:** 2014-05-29

**Authors:** Cinzia Signorini, Silvia Leoncini, Claudio De Felice, Alessandra Pecorelli, Ilaria Meloni, Francesca Ariani, Francesca Mari, Sonia Amabile, Eugenio Paccagnini, Mariangela Gentile, Giuseppe Belmonte, Gloria Zollo, Giuseppe Valacchi, Thierry Durand, Jean-Marie Galano, Lucia Ciccoli, Alessandra Renieri, Joussef Hayek

**Affiliations:** ^1^Department of Molecular and Developmental Medicine, University of Siena, 53100 Siena, Italy; ^2^Child Neuropsychiatry Unit, University Hospital Azienda Ospedaliera Universitaria Senese (AOUS), 53100 Siena, Italy; ^3^Neonatal Intensive Care Unit, University Hospital AOUS, Policlinico “S. M. alle Scotte,” 53100 Siena, Italy; ^4^Medical Genetics, University of Siena, 53100 Siena, Italy; ^5^Department of Life Sciences, University of Siena, 53100 Siena, Italy; ^6^Department of Medicine Surgery and Neuroscience, University of Siena, 53100 Siena, Italy; ^7^Department of Life Science and Biotechnologies, University of Ferrara, 44121 Ferrara, Italy; ^8^Institut des Biomolécules Max Mousseron (IBMM), UMR 5247-CNRS-UM I-UM II-ENSCM, BP 14491 34093, Montpellier Cedex 5, France; ^9^Genetica Medica, Azienda Ospedaliera Universitaria Senese, 53100 Siena, Italy

## Abstract

Evidence of oxidative stress has been reported in the blood of patients with Rett syndrome (RTT), a neurodevelopmental disorder mainly caused by mutations in the gene encoding the Methyl-CpG-binding protein 2. Little is known regarding the redox status in RTT cellular systems and its relationship with the morphological phenotype. In RTT patients (*n* = 16) we investigated four different oxidative stress markers, F_2_-Isoprostanes (F_2_-IsoPs), F_4_-Neuroprostanes (F_4_-NeuroPs), nonprotein bound iron (NPBI), and (4-HNE PAs), and glutathione in one of the most accessible cells, *that is*, skin fibroblasts, and searched for possible changes in cellular/intracellular structure and qualitative modifications of synthesized collagen. Significantly increased F_4_-NeuroPs (12-folds), F_2_-IsoPs (7.5-folds) NPBI (2.3-folds), 4-HNE PAs (1.48-folds), and GSSG (1.44-folds) were detected, with significantly decreased GSH (−43.6%) and GSH/GSSG ratio (−3.05 folds). A marked dilation of the rough endoplasmic reticulum cisternae, associated with several cytoplasmic multilamellar bodies, was detectable in RTT fibroblasts. Colocalization of collagen I and collagen III, as well as the percentage of type I collagen as derived by semiquantitative immunofluorescence staining analyses, appears to be significantly reduced in RTT cells. Our findings indicate the presence of a redox imbalance and previously unrecognized morphological skin fibroblast abnormalities in RTT patients.

## 1. Introduction

Rett syndrome (RTT) predominantly affects females with an incidence of 1 in 10,000–15,000 female births [1, 2]. In its typical form, affected patients exhibit various neuropsychiatric features after 6–18 months [3] of apparently normal neurodevelopment. Although phenotypical heterogeneity which determines the clinical severity of the disease is a typical feature of RTT [4], key clinical aspects include autistic traits, epileptic seizures, breathing abnormalities, gait ataxia, stereotypies, and loss of finalistic hands use [3]. Mutations in the gene encoding the Methyl-CpG-binding protein 2 (*MECP2*) account for approximately 90% of cases with typical RTT and are almost exclusively* de novo* [4]. Nine most frequent mutations comprise more than 3/4 of all the reported pathogenic ones [5]; in addition,* MECP2* gene mutations are usually categorized as missense or truncating, including nonsense, frameshift, and large deletions, according to the type of sequence change.

Oxidative stress (OS) indicates a combination of events resulting in a damage to biological molecules due to an imbalance between cellular antioxidant defences and free radicals production. OS appears to be involved in a large number of human diseases, including cancer [6–8] neurodegenerative diseases [9, 10] and inflammatory bowel disease [11]. Furthermore, cumulating evidence suggests the presence of a redox imbalance in autism spectrum disorders (ASDs), a condition with a high social impact due to the explosive increase in its prevalence over the last four decades [12–18]. Our group and other laboratories have reported enhanced OS markers levels in plasma and erythrocytes from patients with RTT, thus suggesting the presence of a systemic OS in the disease. However, to date, it is unclear not only why, but also when, and where this OS derangement may occur [19–26].

In particular to date, little is known regarding the oxidant-antioxidant status in RTT cellular systems and its possible relationship with the morphological phenotype. Here, we investigated the levels of different OS markers (F_2_-IsoPs, F_4_-NeuroPs, NPBI, and 4-HNE PAs) and glutathione in primary skin fibroblasts from patients with typical RTT, as well as possible changes in the cellular/intracellular structure and/or qualitative changes in the synthesized collagen.

## 2. Materials and Methods

### 2.1. Subjects

A total of 16 female patients with typical RTT (mean age: 13.06 ± 6.5), as well as 8 healthy female controls of comparable age (mean age: 13.2 ± 6.8), participated in the study. Skin biopsies from the control group were obtained from a skin biobank by selecting subjects without diagnosed skin or collagen diseases (responsibility: Joussef Hayek).

All patients were consecutively admitted to the Child Neuropsychiatry Unit of the University Hospital of Siena (Head: Joussef Hayek). For all patients the* MECP2* mutation was demonstrated (mutation categories: missense mutation *n* = 8; early truncating mutation *n* = 6; late truncating mutation *n* = 2), and a clinical severity score was calculated using the previously reported scaling system [27] (mean severity score: 15.9 ± 6.47; range: 5.0–28.0). All the examined subjects were on a typical Mediterranean diet.

A 3 mm skin punch biopsy was performed after obtained written informed consent of either the parents or the legal tutors of the patients (responsibility: Joussef Hayek). Biopsies from control subject were performed in the Dermatology Unit of the University Hospital of Siena. The study was conducted with the approval of the Institutional Review Board of the University Hospital Azienda Ospedaliera Universitaria Senese.

### 2.2. Fibroblasts Isolation and Culture

Following informed consent signature, skin biopsies (about 3-4 mm^3^) were performed using the Punch Biopsy procedure. Fibroblasts were isolated and cultured with standard protocols [28]. Cells were grown in DMEM (Biochrom), supplemented with 10% fetal bovine serum, 100 U/mL penicillin, 100 *μ*g/mL streptomycin, and 2 mM L-glutamine. Cells were incubated at 37°C with 5% CO_2_ until 80–90% confluence and routinely passed by trypsin-EDTA (Irvine Scientific).

Cells for analysis were seeded onto 100 mm tissue culture plates (for NPBI and IsoP evaluations) and 12-well plates (for 4-HNE PAs and GSH/GSSG ratio) containing the indicated medium formulations. Fibroblasts at low passage were employed for the analysis.

At confluence, the cells were scraped, transferred in a tube, and washed twice with ice-cold PBS pH 7.4. Cells were then centrifuged at 600 g for 10 min and the pellet resuspended in a final volume of 2 mL PBS.

### 2.3. Nonprotein Bound Iron (NPBI)

Nonprotein bound iron was determined as desferoxamine- (DFO-) chelatable free iron (DFO-iron complex, ferrioxamine). DFO 25 *μ*M was added to 1 mL of cell suspension to obtain a final concentration of 25 *μ*M DFO and the cells were ruptured by the addition of 1 mL water, freeze-thawing, and sonication. The samples were then ultrafiltered in centrifuge filter devices (VIVASPIN 4, Sartorius Stedim Biotech GmbH, Goettingen, Germany) with a 30 kDa molecular weight cutoff and the ultrafiltrate was stored at −20°C until analysis. The DFO excess was removed by silica (Silicagel 25–40 *μ*m; Merck, Darmstadt, Germany) column chromatography (Varian Inc., CA, USA). The DFO-iron complex was determined by HPLC and the detection wavelength was 229 nm [29].

### 2.4. Total (Sum of Free Plus Esterified) F_2_-Isoprostanes and F_4_-Neuroprostanes Determinations

Butylated hydroxytoluene was added to 900 *μ*L of cell suspension as an antioxidant to obtain a final concentration of 90 *μ*M and the samples were frozen at −70°C until analysis. All isoprostane and neuroprostane determinations were carried out by gas chromatography/negative ion chemical ionization tandem mass spectrometry (GC/NICI-MS/MS) analysis after solid phase extraction and derivatization steps. F_2_-Isoprostanes and F_4_-neuroprostanes levels were normalized for the cell protein content.

### 2.5. Solid Phase Extraction and Derivatization Procedures

At the time of the determination, the samples were sonicated by ultrasound treatment for 30 seconds and then purified as previously reported [29]. Briefly, aqueous KOH (1 mM, 450 *μ*L) was added to the cellular suspension. After incubation at 45°C for 45 min, the pH was adjusted to 3 by adding HCl (1 mM, 450 *μ*L). Each sample was spiked with tetradeuterated prostaglandin F_2*α*_ (PGF_2*α*_-d_4_) (500 pg in 50 *μ*L of ethanol), as an internal standard, and ethyl acetate (10 mL) was added to extract total lipids by vortex-mixing and centrifugation at 1,000 g for 5 min at room temperature. The total lipid extract was applied onto an NH_2_ cartridge and the isoprostanes in the final eluates were derivatized. The carboxylic group was derivatized as the pentafluorobenzil ester whereas the hydroxyl groups were converted to trimethylsilyl ethers [30].

### 2.6. F_2_-Isoprostane GC/NICI-MS/MS

Measured ions were the product ions at* m/z* 299 and* m/z* 303 derived from the [M−181]^−^ precursor ions (*m/z* 569 and* m/z* 573) produced from 15-F_2*t*_-IsoPs and the tetradeuterated derivative of prostaglandin F_2*α*_ (PGF_2*α*_-d_4_), respectively [29, 30].

### 2.7. F_4_-NeuroPs GC/NICI-MS/MS

Measured ions were the product ions at* m/z* 323 and* m/z* 303 derived from the [M−181]^−^ precursor ions (*m/z* 593 and* m/z* 573) produced from oxidized DHA and the tetradeuterated derivative of PGF_2*α*_, respectively [29, 31].

### 2.8. Intracellular Redox Status

Usually, cellular glutathione (GSH) exists mainly in the reduced form whereas the oxidized disulfide form (GSSG) is present in small amounts. The GSH/GSSG ratio is often taken as an indicator of cellular redox status. Intracellular GSH and GSSG levels were determined by an enzymatic recycling procedure according to Tietze [32] and Baker et al. [33]. The SH group of the molecule reacts with 5,5′-dithiobis(2-nitrobenzoic acid) (DTNB), producing a yellow-coloured 5-thio-2-nitrobenzoic acid (TNB), and the disulfide is reduced by NADPH in the presence of GSH reductase. GSSG was determined after derivatization step of GSH by reaction with 2-vinylpyridine. The rate of TNB formation was monitored at 420 nm. GSH and GSSG levels were normalized for protein content.

At confluence, after medium removal, cultured fibroblasts were washed twice with PBS pH 7.4 and treated with 5% 5-sulfosalicylic acid (w/v) solution for 30 min at 4°C. The acidic extracts were stored at −70°C until the assay. The protein extracts were obtained by cellular lysis with NaOH 0.1 M.

### 2.9. 4-HNE Protein Adducts

4-HNE PAs are markers of protein oxidation due to aldehyde binding from lipid peroxidation sources [34], determined by Western blot technique. Cell proteins (30 *μ*g protein, as determined by using Bio-Rad protein assay; BioRad, Hercules, CA, USA) were resolved on 4–20% SDS-PAGE gels (Lonza Group Ltd., Switzerland) and transferred onto a hybond ECL nitrocellulose membrane (GE Healthcare Europe GmbH, Milan, Italy). After blocking in 3% nonfat milk (BioRad, Hercules, CA, USA), the membranes were incubated overnight at 4°C with goat polyclonal anti 4-HNE adduct antibody (cod. AB5605; Millipore Corporation, Billerica, MA, USA). Following washes in TBS Tween and incubation with specific secondary antibody (mouse anti-goat horseradish peroxidase-conjugated, Santa Cruz Biotechnology Inc., CA, USA) for 1 h at room temperature, the membranes were incubated with ECL reagents (BioRad, Hercules, CA, USA) for 1 min. The bands were visualized by autoradiography. Quantification of the relevant bands was performed by digitally scanning the Amersham Hyperfilm ECL (GE Healthcare Europe GmbH, Milan, Italy) and measuring immunoblotting image densities with ImageJ software.

### 2.10. Transmission Electron Microscopy (TEM)

Cultured fibroblasts were fixed in 2.5% glutaraldehyde in 0.1 M cacodylate buffer pH 7.2 (CB) for 3 hours at 4°C. After a rinse in CB, the material was postfixed in 1% osmium tetroxide in CB for 2 hours at 4°C, dehydrated in a graded series of alcohol, embedded in Araldite resins, and polymerized in oven for 48 hours at 60°C. Sixty nm thin sections, obtained with a Reichert ultramicrotome, were routinely stained with uranyl acetate and lead citrate and observed with a TEM Fei Tecnai G2 spirit at 100 Kv.

### 2.11. Immunofluorescence Double Staining

The localization of two main type of collagen synthetized by skin fibroblasts was evaluated by an immunofluorescence assay. Primary human fibroblasts, grown on glass coverslips at a density of 2 × 10^4^ cells/cm^2^, were fixed in 4% paraformaldehyde for 10 min at 4°C. After fixation and permeabilization for 10 min at room temperature with 0.1% Triton X-100, cells were blocked for 30 min at room temperature with PBS containing 5% BSA. Fibroblasts were then incubated with primary antibodies for collagen I and collagen III (Thermo Fisher Scientific Inc., Rockford, IL, USA) in PBS with 1% BSA at 4°C overnight. After washing, cells were incubated with secondary antibodies Alexa Fluor 568 and Alexa Fluor 488 (Life Technologies Corporation, Monza, Italy) for 1 h at room temperature. Nuclei were stained with 1 *μ*g/mL DAPI (Sigma-Aldrich S.r.l., Milan, Italy) for 1 min. Coverslips were mounted onto glass slides using antifade mounting medium 1,4-diazabicyclooctane in glycerine (DABCO). Observations and photographs were made with a Leitz Aristoplan light microscope (Leica, Milan, Italy) equipped with fluorescence apparatus. Incubation in primary antibodies was omitted in negative controls.

The relative intensity of fluorescence was measured in regions of interest (ROI) by using the Software LEICA AF6000 (Leica Microsystems-Germany).

### 2.12. Statistical Analysis

Results were expressed as medians with interquartile ranges or means ± standard deviation. Differences between groups were evaluated by the nonparametric Mann-Whitney rank sum test, Wilcoxon rank test, or Kruskal-Wallis test analysis of variance (ANOVA), as appropriate. The MedCalc ver. 12.1.4 statistical software package (MedCalc. Software, Mariakerke, Belgium) was used for data analysis. Two-sided *P* values <0.05 were considered as significant.

## 3. Results

### 3.1. Cell Oxidant and Antioxidant Status

**Figure 1:**
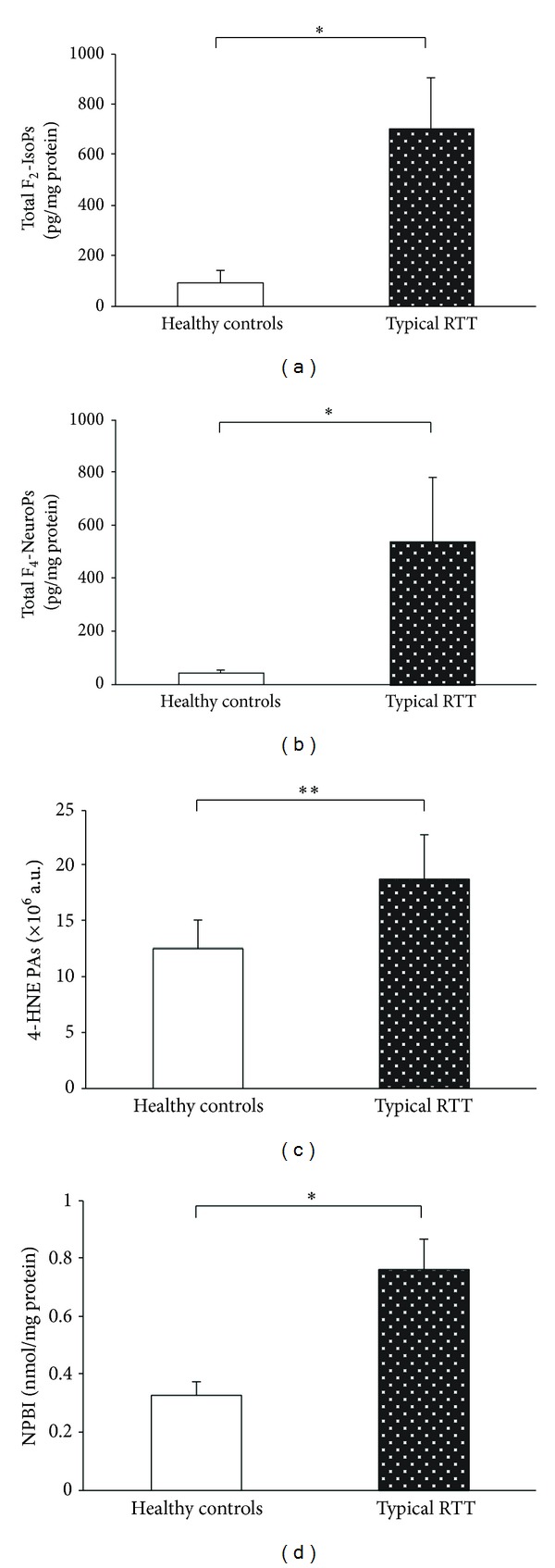
Increased levels of total (i.e., sum of free and esterified form) F_2_-IsoPs, total F_4_-NeuroPs, 4-HNE PAs, and NPBI in RTT skin fibroblast as compared to the control cells. *P < 0.0001,  **P = 0.0013. Data are expressed as means ± standard deviation. Legend: F_2_-IsoPs, F_2_-isoprostanes; F_4_-NeuroPs, F_4_-neuroprostanes; 4-HNE PAs, 4-hydroxy-2-nonenal protein adducts; NPBI, nonprotein bound iron.

**Figure 2:**
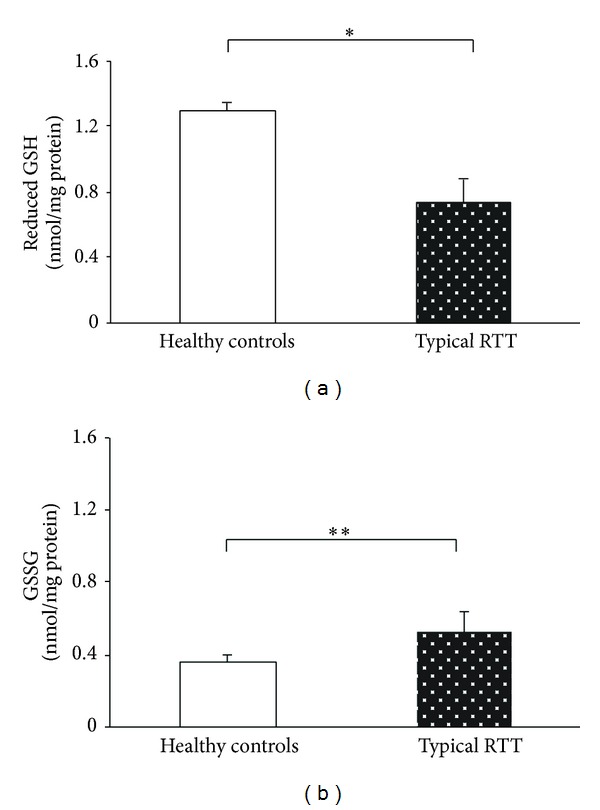
Significant reduction in cellular GSH and significant increase of GSSG in RTT skin fibroblast as compared to control cells. *P < 0.0001,  **P = 0.0033. Data are expressed as means ± standard deviation. Legend: GSH reduced glutathione; GSSG, oxidized glutathione.

Biochemical signs of lipid and protein oxidative damage, together with the antioxidant cellular defense, were evaluated in skin fibroblasts from RTT and healthy control subjects. Increased lipid oxidative damage was evidenced as indicated by increased levels of total (i.e., sum of free and esterified form) F_2_-IsoPs (7.5-folds) and F_4_-NeuroPs (12-folds), both deriving from membrane polyunsaturated fatty acids, that is, arachidonic (AA) and docosahexaenoic (DHA) acid, respectively (Figures 1(a) and 1(b)). Biochemical signs of protein oxidative damage consequent to a lipid peroxidation event were indicated by increased (1.48-folds) 4-HNE PAs levels for which a significant increase was also detected (Figure 1(c)). Oxidative damage was concomitant to an imbalance in the principal antioxidant cytoplasmic agent in so far as a significant reduction (−43.6%) in cellular GSH, a significant increase (1.44-folds) of GSSG (Figures 2(a) and 2(b)), and a significant reduction (−3.05 folds) of GSH/GSSG ratio were reported. Lipid peroxidation events in RTT skin fibroblasts were found to be related to the levels of NPBI, a prooxidant agent. NPBI was significantly increased (2.3-folds) (Figure 1(d)), with significant positive correlations observed between NPBI and total cellular F_4_-NeuroPs (Rho = 0.84, *P* = 0.001) and NPBI* versus* total cellular F_2_-IsoPs (Rho = 0.69, *P* = 0.019). No significant relationships between each of the investigated molecules (i.e., F_2_-IsoPs, F_4_-NeuroPs, 4-HNE Pas, GSH and GSSG) and the* MECP2* mutation categories were observed (*P* value range: 0.461–0.981).

### 3.2. Cell Morphology Study

To evaluate the effect, if present, of the oxidant/antioxidant imbalance evidenced in RTT fibroblasts, the physiological cellular condition was evaluated by observation of two key typical features: the morphology and the collagen distribution.

**Figure 3:**
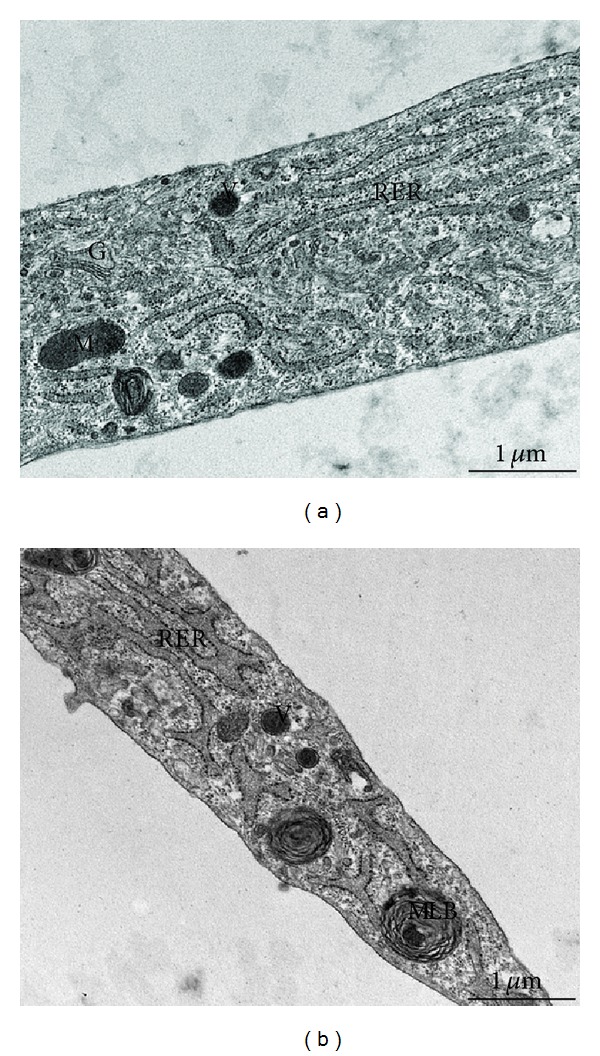
Transmission electron microscopy of control (a) and RTT (b) fibroblasts cultures. Skin fibroblasts, either from control subjects or RTT patients, show a flattened morphology with extensive tapering cytoplasmic processes. An euchromatic and oval-shaped nucleus was present in central position of the cells, with clumps of heterochromatin next to the nuclear envelope. The cytoplasm contains many vesicles with variable electron density, a prominent Golgi complex, and mitochondria. Rough endoplasmic reticulum (RER) cisternae in RTT fibroblasts appear more dilated than in control. Some large multilamellar bodies (MLB) are frequently detectable in the cytoplasm of the RTT fibroblast cells. (G) Golgi complex, (M) mitochondrion, and (V) vesicle. Bar = 1 *μ*m.

**Figure 4:**
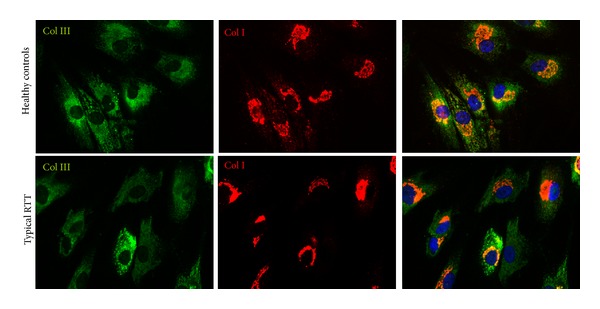
Double immunofluorescence staining shows the localization of type I collagen (central column, red color) and type III collagen (left column, green color). Images are merged in the right panel and the yellow color indicates overlap of the staining. The colocalization of types I and III collagen is reduced in RTT skin fibroblasts. Legend: Col I, type I collagen; Col III, type III collagen.

**Figure 5:**
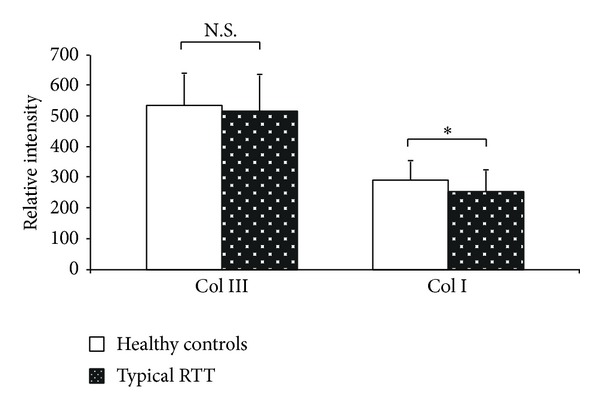
Relative intensity of fluorescence for types I and type III collagen in RTT and control skin fibroblasts. Software LEICA AF6000 (Leica Microsystems-Germany). Data are expressed as median ± semiinterquartile range *P = 0.0062; N.S.: no significant difference (*P* = 0.4361). Legend: Col I, type I collagen; Col III, type III collagen.

At transmission electron microscope significant ultrastructural differences between control and RTT fibroblasts were observed. In particular, a marked dilation of the rough endoplasmic reticulum cisternae was detectable in the skin RTT fibroblasts, along with evidence of cytoplasmic multilamellar bodies (Figures 3(a) and 3(b)). Immunofluorescence double staining showed a reduced degree of colocalization of type III and type I collagen in RTT skin fibroblasts when compared to control cells. Staining for type I collagen was found to be more evident in RTT cells, where perinuclear granules were also detectable (Figure 4). Likewise, in these same cells, fluorescence relative intensity for type I collagen was significantly reduced (*P* = 0.00997) (Figure 5).

## 4. Discussion

Our findings demonstrate, for the first time, the presence of an extensive redox imbalance in primary skin fibroblasts cultures obtained from patients with RTT by adding new evidence to the concept of an OS imbalance as key phenotypical features of MeCP2 deficiency in RTT. Earlier clues for an abnormal OS balance in RTT patients harbouring* MECP2* gene mutations are to date limited to plasma and erythrocytes samples [26, 35]. Although enhanced plasmatic OS markers levels suggest a systemic OS status, to date no indications are present in order to infer what tissues/cellular systems other than blood could be potentially damaged by the OS imbalance in RTT. A preliminary suggestion that an oxidative process should occur at the cellular level was contained in our recent study where oxidative posttranslational modifications on SRB1 receptor in primary fibroblast cultures were observed [36]. Fibroblasts have been used in the RTT scientific research to evaluate gene mutation and epigenetic process [37], differentiation to induced pluripotent stem (iPS) [38], cellular response subsequent to* MECP2* mutation [39], reduction of stathmin-like 2 [40], and the effects of NB54 and other rationally designed aminoglycoside derivatives as potential therapeutic agents for nonsense* MECP2* mutations in RTT [41]. Fibroblasts have been extensively employed to investigate OS in different pathophysiological processes such as genetic neurodegenerative diseases [42], Parkinson's disease [43], aging [44], and response to etiological agents [45, 46]. Moreover, lipid peroxidation events are known to be relevant to the fibroblast function, as relationship between mechanotransduction and lipid metabolites has been previously investigated in keloids [47].

The biochemical markers so far employed for measuring OS are not to be considered as equal in terms of the conveyed information. In particular, F_2_-IsoPs, the gold standard molecules for the OS* in vivo* evaluation [35, 48], are the end-products of arachidonic acid (AA) oxidation, a polyunsaturated fatty acid abundant in both brain grey and white matter. On the other hand, F_4_-NeuroPs are the oxidative end-products of docosahexanoic acid (DHA), abundant in neuronal membranes [49]. NPBI is a prooxidant factor, associated with hypoxia, hemoglobin oxidation, and subsequent heme iron release [50].

Our data demonstrate that both AA and DHA undergo significant oxidation in RTT fibroblasts. Although DHA is known to be particularly abundant in neuronal membranes, this fatty acid represents a normal constituent of all cell membranes. In particular, human fibroblasts are able to synthetize DHA [51] and incorporate it in their membrane phospholipids [52]. Furthermore, variations of the DHA content in the membrane of fibroblasts have been implicated in neuropsychiatric disorders, with lowered levels evidenced in patients with schizophrenia and bipolar disorder [53]. Our results indicate an increased oxidation of DHA in RTT fibroblasts, thus underlying an increased susceptibility of these cells to oxidative damage. This process, in turn, could contribute to changes in the fatty acid composition of membranes with consequent cellular damage.

4HNE, which is formed from arachidonic acid or other unsaturated fatty acids following free radical attack, can bind, by Michael addition, to proteins, particularly, to cysteine, histidine, or lysine residues. Thanks to its ability to form adducts to the proteins, 4HNE is not only considered as a reliable marker of OS, but also has a biological impact by changing protein function [34]. Our findings of increased 4HNE-PAs can be considered as a long-term consequence of enhanced lipid peroxidation, further contributing to cellular damage.

Glutathione, the main cellular antioxidant defence, preventing the damage to key cellular components induced by reactive oxygen species, exists in both reduced (GSH) and oxidized (GSSG) states. In its reduced state, the thiol group of cysteine is able to donate a reducing equivalent (H^+^ + e^−^) to other unstable molecules, such as ROS, whereas in donating an electron, glutathione itself becomes reactive but is ready to react with another reactive glutathione to form GSSG.

Our reported results of decreased GSH levels and GSH/GSSG ratio indicate a reduced antioxidant defence in RTT cells and suggest that oxidative events are likely to be chronic, thus determining a consumption of glutathione in its reduced form. An alternative explanation could be that the* MECP2* mutation-harboring cells may have coexisting defects in the synthesis and/or recycling of glutathione, thus leading to an uncontrolled free radicals action. Of course, further study is needed in order to clarify this point.

Overall, our data indicate that a significant oxidation of DHA, the precursor acid for F_4_-NeuroPs, and AA, precursor of F_2_-IsoPs, likely triggered by NPBI, occurs in skin fibroblasts of patients with* MECP2* mutation and clinical RTT and confirm the biological relevance of such key mediators of lipid peroxidation.

Skin dermis is known to be consisting of 80% collagen type I, with its remaining fraction being made mostly of collagen type III [54]. In this context, the fibroblast shows a pivotal role for collagen production, [55].

Besides being a major component of the extracellular matrix in a variety of internal organs and skin in adults, type III collagen is critical for fibrillogenesis in the development of apparatus such as skin and cardiovascular system [56, 57]. Overall in such tissues, type III collagen is colocalized, within the same fibril, with the most abundant member of the family, type I collagen, and regulates the diameter of type I collagen fibrils, which have to meet the physiological requirements of different tissues at different developmental stages [54, 58–62].

The reported ultrastructural changes are to be considered as a new finding to be added to the mitochondrial changes previously reported in RTT skin fibroblasts [63]. Since no abnormalities in wound repair and/or healing have been reported in RTT patients, these ultrastructural features are to be considered as subclinical characteristics of the disease. Marked dilation of the rough endoplasmic reticulum cisternae and cytoplasmic multilamellar bodies were observed for the first time in this study. Dilated rough endoplasmic reticulum cisternae can be considered as a nonspecific adaptive response due to either increased secretory activity or as the result of the cellular response to noxae of different nature [64, 65]. On the other hand, the presence of multilamellar bodies could be interpreted as an expression of cellular damage. In particular, those structures suggest the occurrence of autophagy phenomena of unclear pathogenesis [66].

A condition of oxidative stress has been previously reported in aging skin [67]. Skin aging is known to be mainly due to fragmentation/loss of type I collagen fibrils, conferring strength, and resiliency in association with metalloproteinase activation, involved in type I collagen degradation [68]. Although, no evidence of wound healing alterations or derma laxity has not been reported in RTT, an accelerated ageing process is known to occur in the affected patients. Our results suggest the occurrence of a possible reduction in type I collagen, a feature of aging skin, while they show a clear redox imbalance in RTT skin fibroblasts. Therefore, it is conceivable that a premature skin aging may occur in RTT patients, a hypothesis which is in line with prior evidence of premature senescence phenomena in the disease [69–72].

Given that OS is known to be a major determinant of aging [73, 74], it is conceivable that aging phenomena in RTT, including skin fibroblasts morphological changes, may be caused through OS mechanisms.

## 5. Conclusion

Our study demonstrates for the first time the occurrence of lipid peroxidation in RTT fibroblasts, together with a reduced antioxidant cellular defense with a major impact on cell morphology. We speculate about a possible functional involvement of these changes in the skin fibroblasts of RTT patients which must be taken into account when evaluating this cellular model of the disease. Our findings suggest that OS is a generalized phenomenon in RTT, thus affecting cellular systems and tissues apparently unrelated to the central nervous system.

## Abbreviations

4-HNE: 4-Hydroxy-2-nonenal
4-HNE PAs: 4-Hydroxy-2-nonenal protein adducts
AA: Arachidonic acid
DHA: Docosahexaenoic acid
F_2_-IsoPs: F_2_-isoprostanes
F_4_-NeuroPs: F_4_-neuroprostanes
GSH: Reduced glutathione
GSSG: Oxidized glutathione
IsoPs: Isoprostanes
*MECP2*: Methyl-CpG-binding protein 2—human gene
MeCP2: Methyl-CpG-binding protein 2—human protein
NPBI: Nonprotein bound iron
OS: Oxidative stress
PUFAs: Polyunsaturated fatty acids
ROS: Reactive Oxygen Species
RTT: Rett syndrome
TEM: Transmission electron microscopy.
